# Prognostic biomarkers correlated with immune infiltration in non‐small cell lung cancer

**DOI:** 10.1002/2211-5463.13501

**Published:** 2022-11-28

**Authors:** Fei Xu, Wen‐qiang Cui, Cun Liu, Fubin Feng, Ruijuan Liu, Jingtao Zhang, Chang‐gang Sun

**Affiliations:** ^1^ Department of Geriatric Medicine Affiliated Hospital of Shandong University of Traditional Chinese Medicine Jinan China; ^2^ First Clinical Medical College Shandong University of Traditional Chinese Medicine Jinan China; ^3^ Department of Neurology Affiliated Hospital of Shandong University of Traditional Chinese Medicine Jinan China; ^4^ College of Traditional Chinese Medicine Shandong University of Traditional Chinese Medicine Jinan China; ^5^ Department of Oncology Weifang Traditional Chinese Hospital China; ^6^ Qingdao Academy of Chinese Medical Sciences Shandong University of Traditional Chinese Medicine Qingdao China

**Keywords:** lung cancer, next‐generation sequencing, non‐small cell lung cancer, prognosis, tumor mutation burden, tumor immune microenvironment

## Abstract

Lung cancer is the leading cause of cancer‐related mortality in men and women globally. Non‐small cell lung cancer (NSCLC) is the most prevalent subtype, accounting for 85–90% of all cancers. Although there have been dramatic advances in therapeutic approaches in recent decades, the recurrence and metastasis rates of NSCLC are as high as 30–40% with the 5‐year overall survival rate being less than 15%. Therefore, it is necessary to explore the pathogenesis of NSCLC at the genetic level and identify prognostic biomarkers and novel therapeutic targets. Here, we aimed to identify mutated genes with high frequencies in Chinese NSCLC patients using next‐generation sequencing and to investigate their relationships with the tumor mutation burden (TMB) and tumor immune microenvironment. A total of 110 NSCLC patients were enrolled to profile the genetic variations. Mutations in *EGFR* (62.37%), TP53 (61.29%), *LRP1B* (13.98%), *FAT1* (12.90%), *KMT2D* (11.83%), *CREBBP* (10.75%), and *RB1* (9.68%) were most prevalent. *TP53*, *LRP1B*, *KMT2D*, and *CREBBP* mutations were all significantly associated with high TMB (*P* < 0.05 or *P* < 0.01). The infiltrating levels of immune cells and immune molecules were enriched significantly in the LRP1B mutation group. LRP1B mutations significantly correlated with stimulating and inhibitory immunoregulators. Gene set enrichment analysis revealed that cell cycle, the Notch signaling pathway, the insulin signaling pathway, and the mTOR signaling pathway are related to LRP1B mutations in the immune system. LRP1B mutations may be of clinical importance in enhancing the anti‐tumor immune response and may be a promising biomarker for predicting immunotherapy responsiveness.

## Graphical abstract

LRP1B mutations are significantly associated with prognosis and with a higher tumor mutation burden in non‐small cell lung cancer (NSCLC). The infiltrating levels of immune cells and immune molecules are enriched significantly in the LRP1B mutation group. LRP1B mutations are significantly correlated with some stimulating and inhibitory immunoregulators. LRP1B may be a promising biomarker for predicting immunotherapy responsiveness for NSCLC.

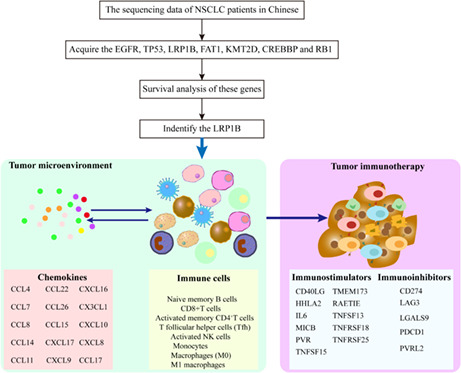

**Abbreviations**
FDR: false discovery rate
GSEA: gene set enrichment analysis
HCC: hepatocellular carcinoma
ICIs: immune checkpoint inhibitors
IICs: infiltrated immune cells
LUAD: lung adenocarcinoma
LUSC: lung squamous cell carcinoma
MSI: microsatellite instability
NGS: next‐generation sequencing
NSCLC: non‐small cell lung cancer
PD‐1: programmed cell death protein 1
PD‐L1: programmed cell death protein ligand 1
TILs: tumor‐infiltrating lymphocytes
TMB: tumor mutation burden

Lung cancer is the most prevalent malignancy and the leading cause of cancer‐related mortality in both men and women, worldwide [1]. Non‐small cell lung cancer (NSCLC), as the most prevalent subtype, including lung adenocarcinoma (LUAD) and lung squamous cell carcinoma (LUSC), accounts for 85–90% of all cancers. Although there have been dramatic advances in therapeutic approaches in recent decades, the recurrence and metastasis rates of NSCLC are as high as 30–40% with the 5‐year overall survival rate being less than 15% [2]. The cellular and molecular mechanisms underlying the pathogenesis of NSCLC are driven by multiple genes, including oncogenes and tumor suppressor genes. Therefore, it is necessary to explore the pathogenesis of NSCLC at the genetic level and identify prognostic biomarkers and novel therapeutic targets.

Cancer immunotherapy is a major revolution in tumor therapy. Immune checkpoint inhibitors (ICIs), such as monoclonal antibodies targeting programmed cell death protein 1 (PD‐1), programmed cell death protein ligand 1 (PD‐L1), or cytotoxic T cell antigen 4, are utilized as first‐line therapies for a variety of solid and liquid cancers, including NSCLC. The use of ICIs can improve survival and quality of life greatly, and sometimes result in long‐term disease control. However, only a small percentage of the patients responded well to ICIs [3]. A large number of patients achieved primary or secondary immunotherapy resistance with unpromising outcomes. Although effort has been put into searching for a variety of biomarkers for the unique sensitivity of ICI therapy, three have been approved by the US Food and Drug Administration with some limitations, including microsatellite instability (MSI), PD‐L1, and tumor mutation burden (TMB).

A large body of evidence has shown that genetic alterations are linked intrinsically to immune infiltrates and immune response [4, 5, 6]. The specific activation of the proto‐oncogenes or loss of function of the tumor suppressor genes enhances or dampens antitumor immunity [7]. Next‐generation sequencing (NGS) is being used increasingly in clinical pathology laboratories to capture the numerous mutations in multiple specific genomic regions [8]. Special and clinically impactful variants can be identified, which provide clues for discovering targetable and prognosticative genes, clarifying the mechanism, and novel therapeutic approaches. Recent studies have identified specific molecular alterations using NGS and have demonstrated connections between their status, immune infiltration, and prognosis in different cancers [9, 10].

In this study, we analyzed the genetic aberrations in NSCLC patients using targeted NGS, examined their potential association with immune cell infiltration, identified patients who may benefit from ICI therapy, and evaluated their prognostic values.

## Materials and methods

### Patients

In total, 126 patients with histologically confirmed NSCLC were enrolled in this study (Table 1). Patients were excluded if they had received targeted drugs or ICIs. Written informed consent was obtained from all the patients who participated in this study. The clinical information was extracted from the electronic medical records. This study conformed to the standards set by the Declaration of Helsinki and was approved by the Ethics Committee of the Weifang Traditional Chinese Hospital (Approval No. 2021‐WFRS‐006).

**Table 1:** Characteristics of NSCLC patients.

Sample ID	Gender	Age	Clinical diagnosis	Specimen
P1	Male	64	NSCLC	Blood
P2	Male	59	NSCLC	Blood
P3	Male	58	NSCLC	Blood
P4	Female	64	LUAD	Blood
P5	Female	64	LUAD	Blood
P6	Male	53	LUAD	Blood
P7	Male	58	LUAD	Blood
P8	Male	58	NSCLC	Blood
P9	Female	69	NSCLC	Blood
P10	Male	63	LUAD	Blood
P11	Male	65	NSCLC	Blood
P12	Female	64	NSCLC	Blood
P13	Male	40	NSCLC	Blood
P14	Female	49	NSCLC	Blood
P15	Female	68	LUAD	Blood
P16	Male	65	NSCLC	Blood
P17	Male	66	LUAD	Blood
P18	Male	65	NSCLC	Blood
P19	Female	70	LUAD	Blood
P20	Male	62	LUAD	Blood
P21	Female	63	LUAD	Blood
P22	Male	52	LUAD	Blood
P23	Male	68	LUAD	Blood
P24	Male	82	NSCLC	Blood
P25	Male	69	LUAD	Blood
P26	Male	44	LUAD	Blood
P27	Female	55	LUSC	Blood
P28	Female	52	LUSC	Blood
P29	Female	53	LUAD	Blood
P30	Male	71	NSCLC	Tissue
P31	Female	81	LUAD	Tissue
P32	Male	51	NSCLC	Tissue
P33	Male	67	NSCLC	Tissue
P34	Female	62	NSCLC	Tissue
P35	Male	85	LUAD	Tissue
P36	Male	55	NSCLC	Tissue
P37	Male	65	NSCLC	Tissue
P38	Male	55	LUAD	Tissue
P39	Male	61	NSCLC	Tissue
P40	Female	64	NSCLC	Tissue
P41	Female	57	NSCLC	Tissue
P42	Male	56	LUSC	Tissue
P43	Male	65	NSCLC	Tissue
P44	Male	61	NSCLC	Tissue
P45	Female	64	NSCLC	Tissue
P46	Female	57	NSCLC	Tissue
P47	Male	56	LUSC	Tissue
P48	Male	65	NSCLC	Tissue
P49	Male	56	LUAD	Tissue
P50	Female	56	LUAD	Tissue
P51	Male	71	NSCLC	Tissue
P52	Male	77	NSCLC	Tissue
P53	Male	52	LUSC	Tissue
P54	Female	45	LUAD	Tissue
P55	Male	66	LUSC	Tissue
P56	Male	66	LUSC	Tissue
P57	Male	67	NSCLC	Tissue
P58	Male	76	NSCLC	Tissue
P59	Male	62	LUAD	Tissue
P60	Female	62	LUAD	Tissue
P61	Male	70	LUAD	Tissue
P62	Male	67	LUSC	Tissue
P63	Female	70	LUAD	Tissue
P64	Male	57	LUAD	Tissue
P65	Female	60	LUSC	Tissue
P66	Male	47	LUAD	Tissue
P67	Male	67	LUSC	Tissue
P68	Male	64	LUAD	Tissue
P69	Male	66	LUAD	Tissue
P70	Male	74	LUAD	Tissue
P71	Female	67	LUAD	Tissue
P72	Male	51	LUAD	Tissue
P73	Female	64	LUAD	Tissue
P74	Male	69	LUAD	Tissue
P75	Male	61	LUSC	Tissue
P76	Female	64	LUAD	Tissue
P77	Male	48	LUAD	Tissue
P78	Male	64	LUAD	Tissue
P79	Female	64	LUAD	Tissue
P80	Female	47	LUSC	Tissue
P81	Male	65	LUSC	Tissue
P82	Male	67	LUSC	Tissue
P83	Female	47	LUAD	Tissue
P84	Female	45	LUAD	Tissue
P85	Male	51	LUAD	Tissue
P86	Female	68	LUSC	Tissue
P87	Female	64	LUAD	Tissue
P88	Male	51	LUAD	Tissue
P89	Female	67	LUAD	Tissue
P90	Female	54	LUAD	Tissue
P91	Female	70	LUSC	Tissue
P92	Male	75	LUAD	Tissue
P93	Male	74	LUAD	Tissue
P94	Female	67	LUAD	Tissue
P95	Male	62	LUAD	Tissue
P96	Male	53	LUAD	Tissue
P97	Female	63	LUSC	Tissue
P98	Male	61	LUAD	Tissue
P99	Male	56	LUAD	Tissue
P100	Female	78	LUAD	Tissue
P101	Female	66	LUAD	Tissue
P102	Male	69	LUAD	Tissue
P103	Female	61	LUSC	Tissue
P104	Female	50	LUAD	Tissue
P105	Male	62	LUAD	Tissue
P106	Female	59	LUAD	Tissue
P107	Female	56	LUAD	Tissue
P108	Male	51	LUAD	Tissue
P109	Female	64	LUAD	Tissue
P110	Female	62	LUAD	Tissue

### Next‐generation targeted sequencing

All the specimens were tested using Sinotech Genomics (Shanghai, China). Genomic DNA originating from tissues or blood was prepared for targeted NGS using a panel of 593 cancer‐related genes. Information, including gene mutations (frameshift, nonsense, missense, or splice site), TMB, and MSI, was captured and described.

### Bioinformatic analysis

#### cBioPortal

The cBioPortal (http://www.cbioportal.org) is a user‐friendly website that provides cancer genomics datasets [11]. For NSCLC, the genomic data of 5604 patients from 6008 samples in 18 studies were selected. Using the data from cBioPortal, we analyzed the genetic alterations, copy number alterations, and correlations between the gene status and prognosis.

#### TCGA

The TCGA (https://portal.gdc.cancer.gov/) is an open‐access resource for various types of cancers. The clinical data for NSCLC (LUAD and LUSC) and normal samples were downloaded from the TCGA. The patients were divided into two groups according to their gene expression levels and mutation status. All the data preprocessing and analyses were conducted using strawberry perl and r software. The “limma” package was used to visualize the extracted data. The r software survival package was used for visualization, and the Kaplan–Meier survival curve was obtained. The relative abundance of infiltrated immune cells (IICs) was investigated using CIBERSORT, a deconvolution algorithm (https://cibersort.stanford.edu/).

#### TISIDB

TISIDB (http://cis.hku.hk/TISIDB/index.php) was used for seeking the correlation between gene status and immune system. We created the landscape of association between gene status and immunostimulators/immunoinhibitors across multiple cancer types via “Immunomodulator” module. Additionally, the correlation between gene status and chemokines/chemokine receptors across multiple cancer types via “chemokine” module was also investigated based on TISIDB database. Meanwhile, the appropriate molecular with the statistical significance of Spearman's correlation was further explored.

### Gene set enrichment analysis

Based on TCGA data, the function of LRP1B mutation and its related signaling pathways were explored through Broad Institute gsea software 3.0. We evaluated the importance of the association between gene sets and functional enrichment/pathways according to the normalized enrichment score, nominal *P*‐value, and false discovery rate (FDR) q‐value.

### Statistical analysis

The spss 19.0 and graphpad prism 7.0 software were used for the analyses. The differences between the two groups were assessed using a nonparametric test. Survival analyses were performed using the log‐rank test. A Spearman correlation analysis was used to calculate the correlations between the infiltrating immune cells. *P* value was less than 0.05 was considered to be statistically significant.

## Results

### Patient characteristics

**Fig. 1:**
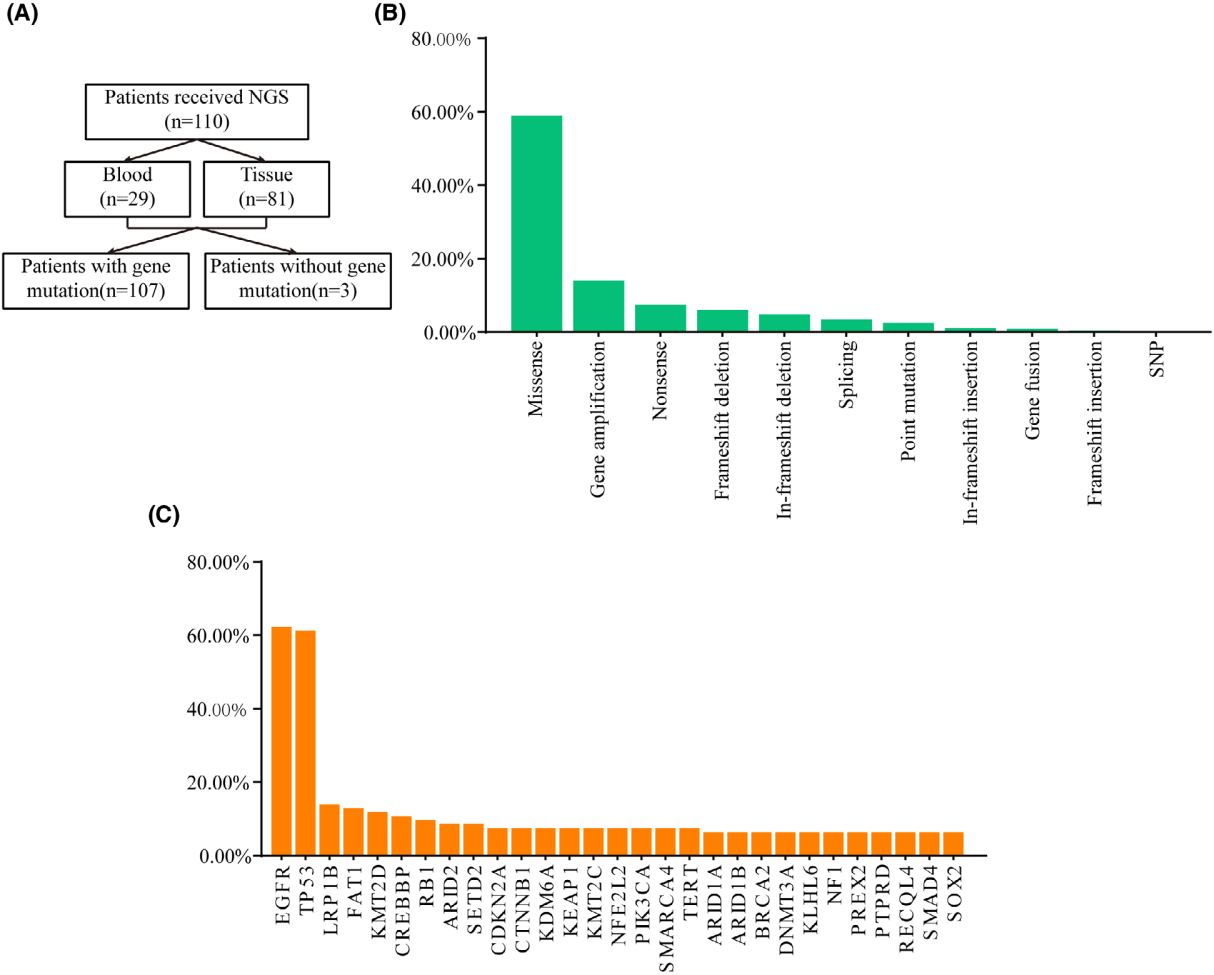
Genomic alternations of the Chinese patients with NSCLC tested by NGS. (A) The flowchart of this study. (B) Distribution of mutation type in NSCLC samples. (C) The frequency of main genetic alterations identified in NSCLC samples.

In the present study, we conducted a retrospective analysis of 110 patients with NSCLC who underwent NGS. A total of 29 blood and 81 tissue samples were collected for further study (Fig. 1A). The median age was 61.74 ± 8.485 years (range, 40–85 years). There were 67 men (60.9%) and 43 women (39.1%). Details of the individual clinical information are shown in Table 1.

### Somatic mutation characteristics

Of the 110 NSCLC specimens, gene mutations in three were not measured (Fig. 1A). There were 788 mutations in all 107 samples and the dominant type was a missense mutation (Fig. 1B). Mutagenesis occurred primarily in the purines, and G > A/G > T was the dominant substitution type (data not shown). The top 29 mutated genes identified in NSCLC patients with high mutation frequencies are shown in Fig. 1C. Among these, the seven most frequently mutated genes were EGFR (62.37%), TP53 (61.29%), LRP1B (13.98%), FAT1 (12.90%), KMT2D (11.83%), CREBBP (10.75%), and RB1 (9.68%) (Fig. 1C).

**Fig. 2:**
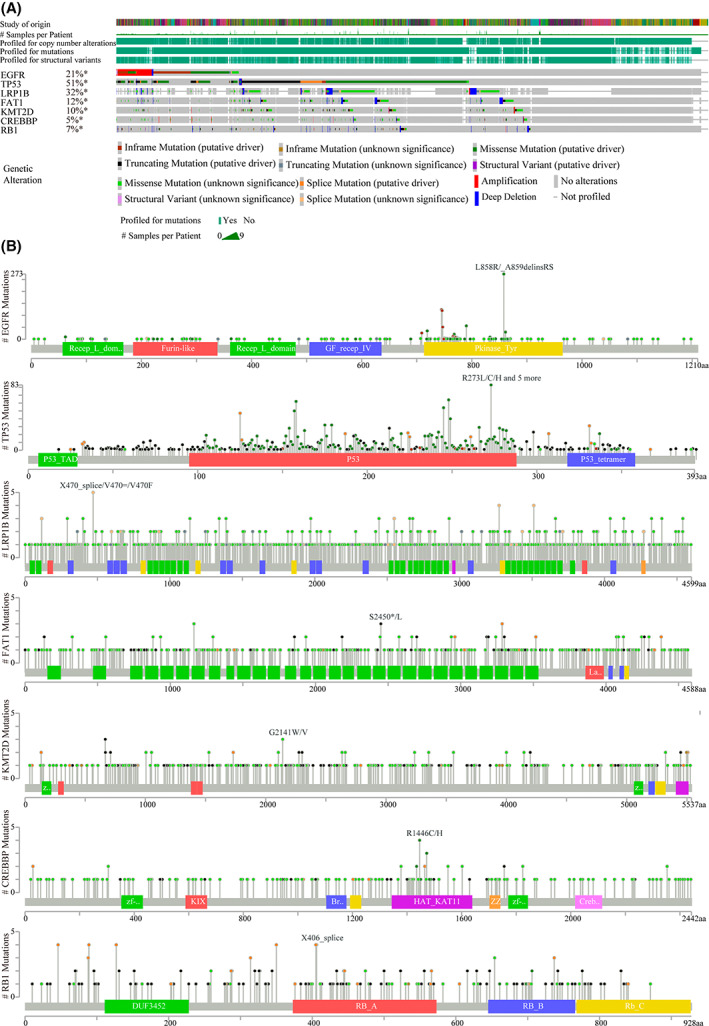
Gene mutational landscape in NSCLC patients from TCGA cohort by using cBioPortal network tool. (A) The mutation frequencies of EGFR, TP53, LRP1B, FAT1, KMT2D, CREBBP, and RB1 in NSCLC from TCGA cohort. (B) The mutant sites of the above seven genes in NSCLC from TCGA cohort.

We also analyzed the frequencies of the above seven mutated genes in the NSCLC samples from the TCGA cohort using the cBioPortal network tool. As shown in Fig. 2A, the frequencies were 21% (EGFR), 47% (TP53), 30% (LRP1B), 11% (FAT1), 9% (KMT2D), 5% (CREBBP), and 6% (RB1), respectively. Missense mutations were the most common type of NSCLC (Fig. 2A), which was consistent with our results. Mutation mapper analysis indicated that the mutant sites of these seven genes with higher mutation frequencies were L858R/_A859delinsRS (EGFR), R273L/C/H, and five more (TP53), X470_splice/V470=/V470F (LRP1B), S2450*/L (FAT1), G2141W/V (KMT2D), R1446C/H (CREBBP), and X406_splice (RB1), respectively (Fig. 2B).

In summary, EGFR, TP53, LRP1B, FAT1, KMT2D, CREBBP, and RB1 from our samples were also frequently mutated genes in the TCGA cohort. They were then chosen for subsequent analysis.

### Patient characteristics and gene mutations associated with the TMB

**Fig. 3:**
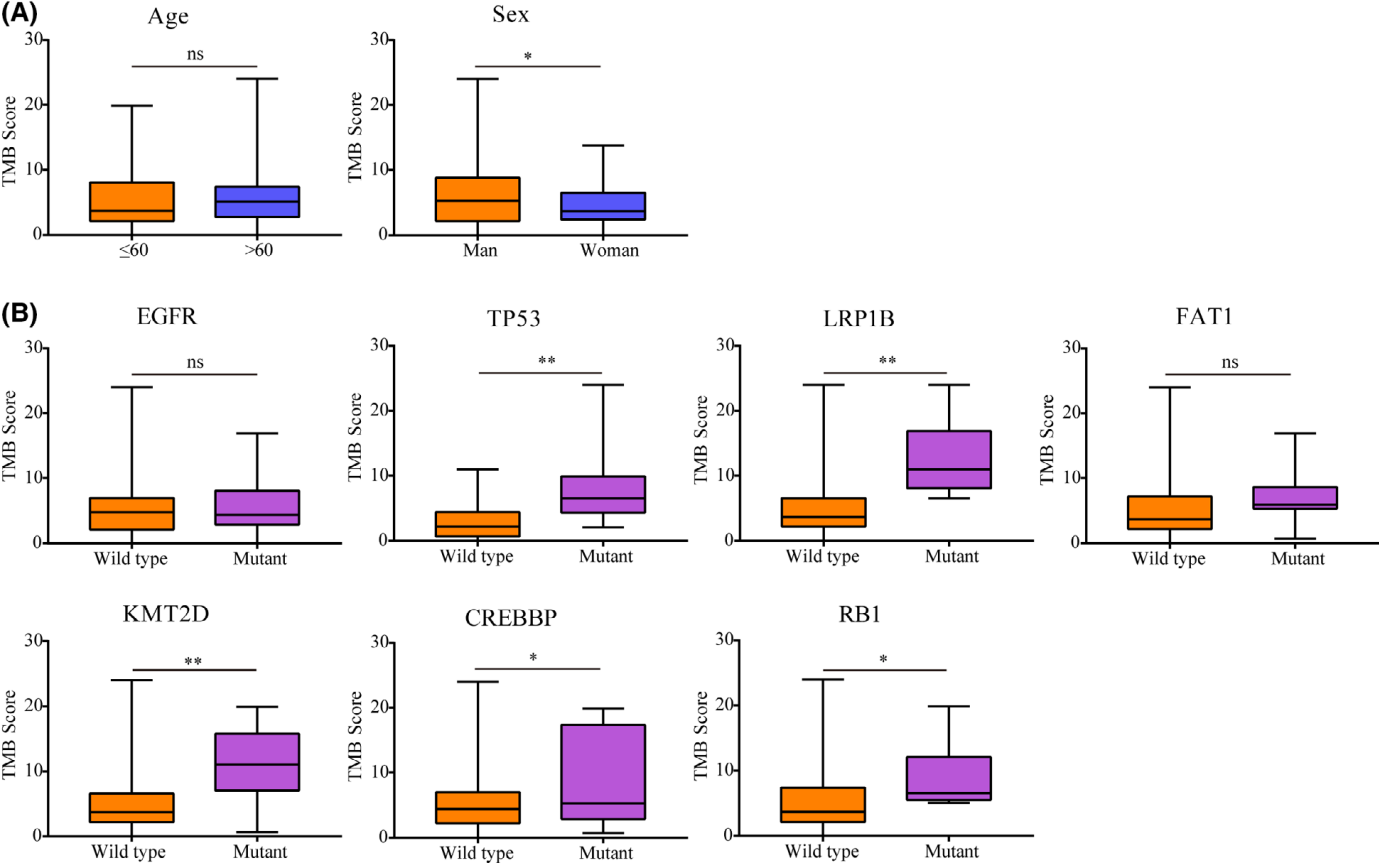
Association of TMB with clinical features and gene mutation. (A) Significant difference was observed in age groups, not gender groups. (B) Most gene mutations were associated with a higher TMB, except for EGFR and FAT1. Ns, *P* ≥ 0.05; *P < 0.05; **P < 0.01.

Tumor mutation burden is an emerging biomarker for identifying patients who may benefit from immunotherapy. Associations between TMB, patient characteristics, and gene status were explored in this study. The median TMB was 4.40 mutations/Mb (ranging, 0–24 mutations/Mb). No significant difference was observed in TMB scores between the two age groups (*P* > 0.05, Fig. 3A). However, TMB scores were significantly higher in the male group (*P* < 0.05, Fig. 3A). Samples with TP53, LRP1B, KMT2D, and CREBBP mutations also showed an obvious increase in TMB scores compared with those without gene mutations (all *P* < 0.05, Fig. 3B). Nevertheless, the TMB values in the EGFR and FA1T mutation groups were not significantly different from those in the wild‐type group (both *P* > 0.05, Fig. 3B).

### Associations between LRP1B gene mutation and prognosis values

**Fig. 4:**
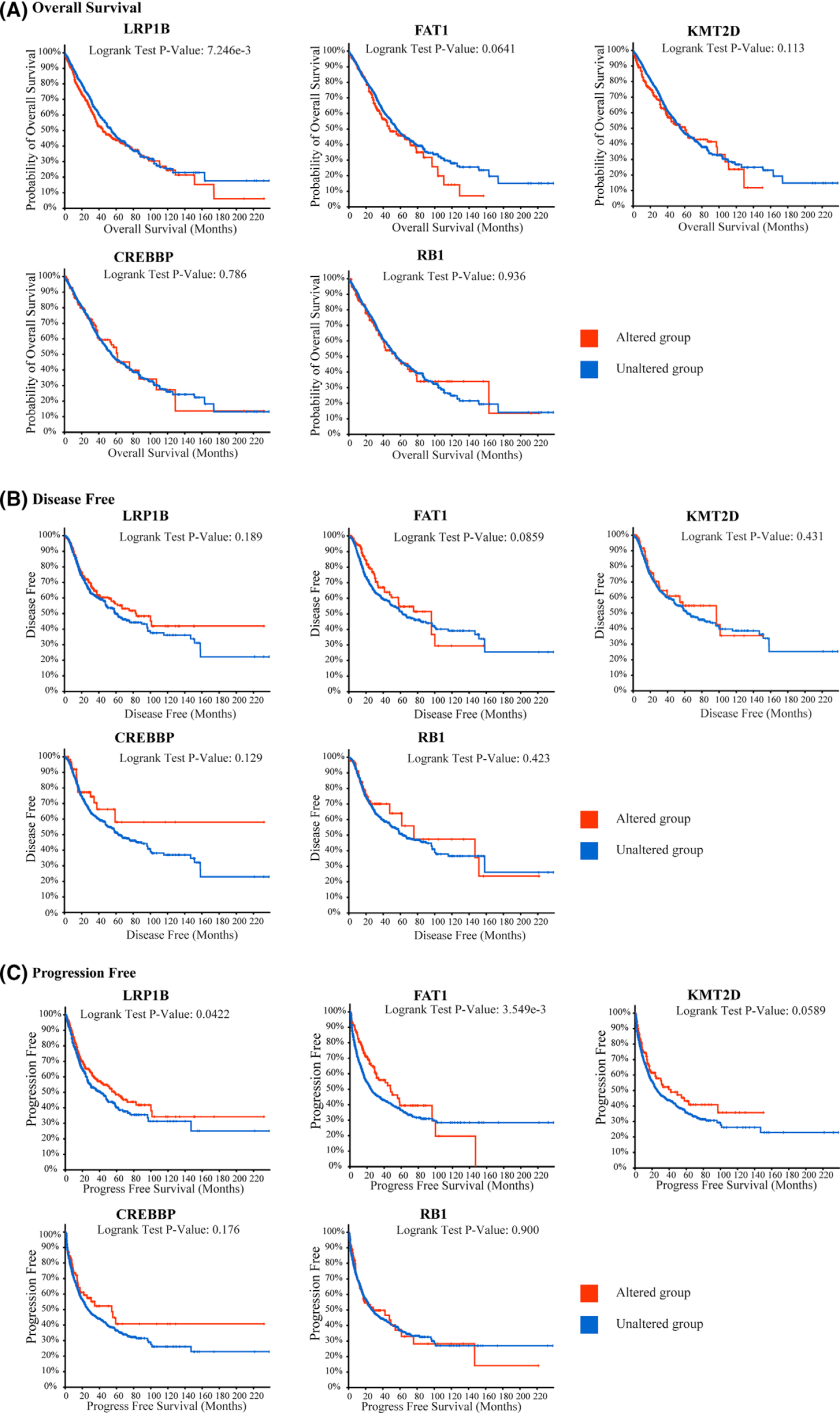
The gene mutation and survival analysis in NSCLC samples from TCGA cohort by using cBioPortal network tool. Kaplan–Meier survival curves showed the predictive value of LRP1B, FAT1, KMT2D, CREBBP and RB1 mutation for overall survival (A), disease‐free survival (B) and PFS (C) in the NSCLC patients.

Correlations between the different gene statuses and survival outcomes were analyzed using cBioPortal. The OS rate was significantly lower in the LR1B altered group (*P* < 0.01, Fig. 4A); however, the progression‐free survival (PFS) benefit was more prominent in the LRP1B‐altered group than in the LRP1B‐unaltered group (*P* < 0.05, Fig. 4C). Nevertheless, these two conclusions are contradictory. Moreover, the OS and disease‐free survival (DFS) differences between the subgroups according to the FAT1, KMT2D, CREBBP, and RB1 altered status were not significant, including the DFS difference between the two subgroups of LRP1B (all *P* > 0.05, Fig. 4A,B). Additionally, the NSCLC patients with FAT1 alterations had a better PFS (*P* < 0.01, Fig. 4C).

### Association of LRP1B gene mutation and immune cell infiltration

**Fig. 5:**
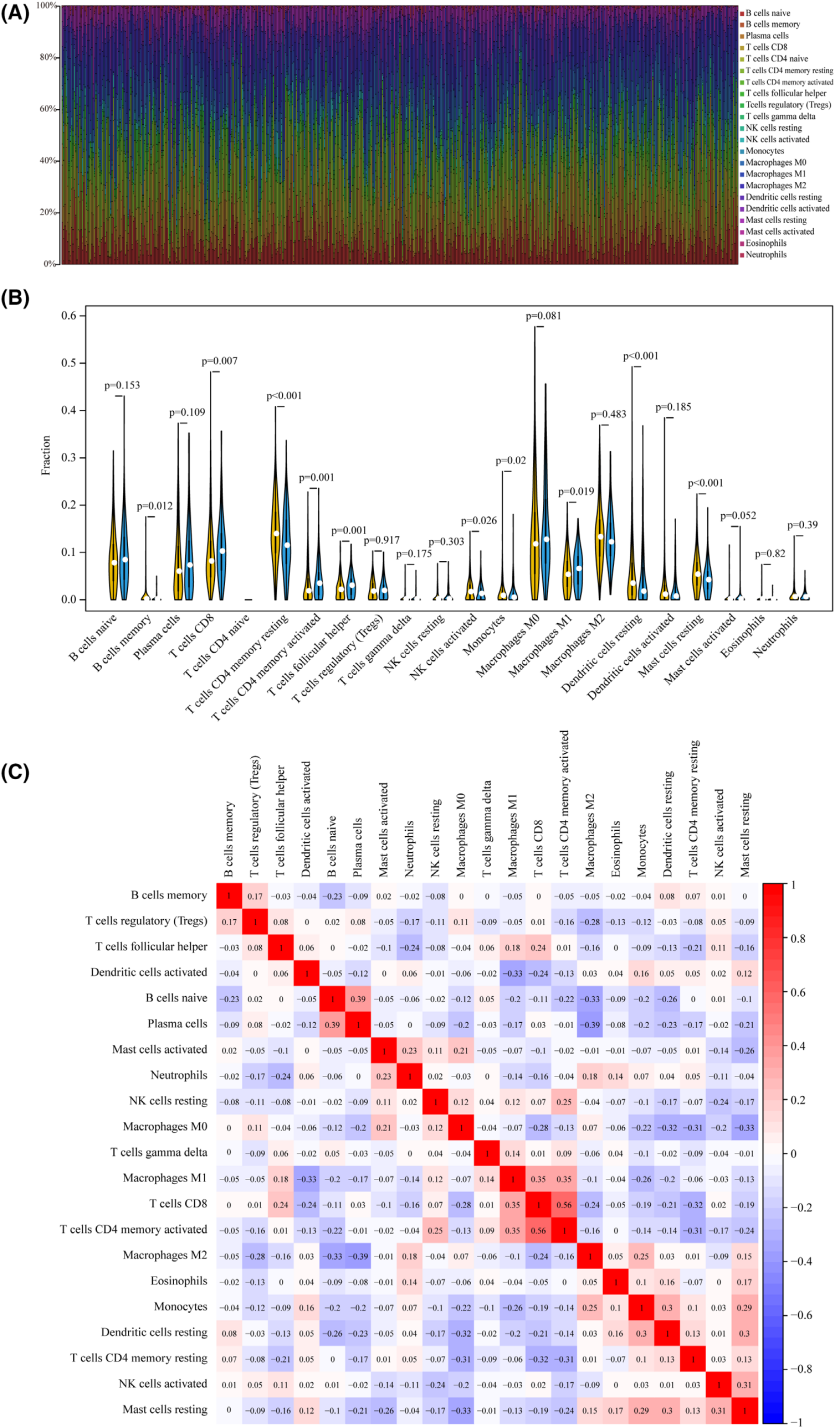
LPR1B mutation is correlated with tumor‐infiltrating immune cells in LUAD. (A) the 22 immune cells in each LUAD sample with LRP1B mutation were annotated by stacked bar chart using the CIBERSORT algorithm. (B) Violin plot for the different proportions of tumor‐infiltrating immune cells between LRP1B‐mutant groups and LRP1B‐wild groups in LUAD. Yellow color represents LRP1B‐wild group, and blue color represents LRP1B‐mutant group. (C) Correlation matrix of 22 types of fractions of tumor‐infiltrating immune cell in LUAD. The red color represents positive correlation and the blue color represents negative correlation.

**Fig. 6:**
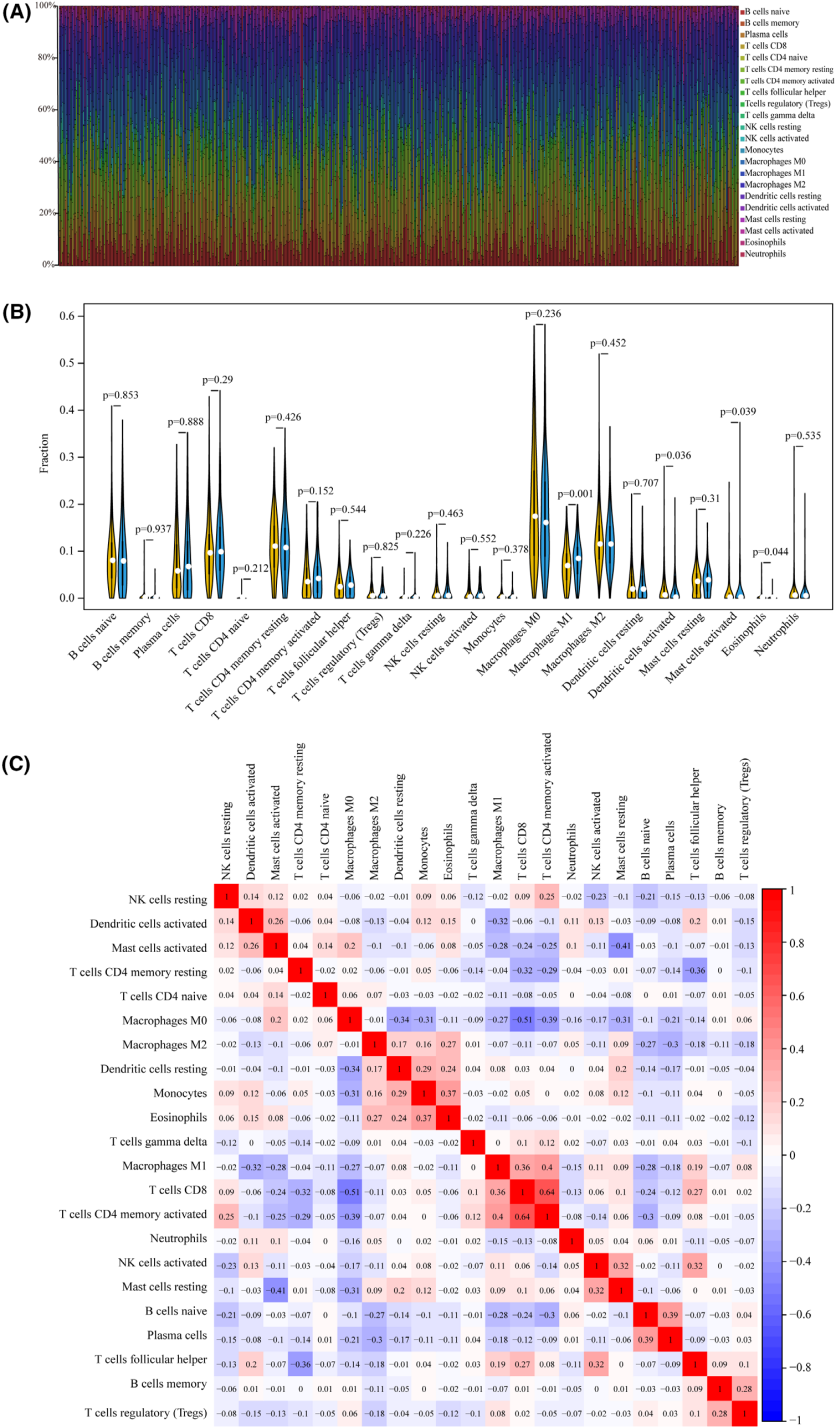
LPR1B mutation is correlated with tumor‐infiltrating immune cells in LUSC. (A) the 22 immune cells in each LUSC sample with LRP1B mutation were annotated by stacked bar chart using the CIBERSORT algorithm. (B) Violin plot for the different proportions of tumor‐infiltrating immune cells between LRP1B‐mutant groups and LRP1B‐wild groups in LUSC. Yellow color represents LRP1B‐wild group, and blue color represents LRP1B‐mutant group. (C) Correlation matrix of 22 types of fractions of tumor‐infiltrating immune cell in LUSC. The red color represents positive correlation and the blue color represents negative correlation.

Given the above results, especially because the LRP1B mutation serves as a strong negative prognostic biomarker for patients, the gene LRP1B was chosen for the next study. We used the CIBERSORT deconvolution algorithm to uncover the association between the LRP1B mutations and the 22 tumor‐infiltrating immune cells in LUAD and LUSD patients. Figs 5A and 6A showed the distribution of IICs in each sample. The results depicted in the integrated heatmap revealed that the abundance of immune cells in LUAD and LUSD varied greatly in each sample. Compared to those of other immune cells, the fractions of T cells, NK cells, and macrophages were relatively higher in the total samples (Figs 5A and 6A).

In TCGA samples, for LUAD, naive memory B cells, CD8^+^T cells, activated memory CD4^+^T cells, T follicular helper cells (Tfh), activated NK cells, monocytes, macrophages (M0), and M1 macrophages were more enriched in the LRP1B mutant type group; however, the memory resting CD4^+^T cells, M2 macrophages, and resting mast cells were enriched in the wild‐type group (Fig. 5B). Furthermore, the correlation analysis of IICs revealed that the activated memory CD4^+^T cells were associated strongly and positively with the CD8^+^T cells, γδ T cells, resting NK cells, and M1 macrophages but negatively correlated with the Tregs (Fig. 5C). For LUSD, while was a more noticeable increase in the M1 macrophage and eosinophil cells infiltration of the LRP1B mutation than in the wild type, there was a significant decrease in the fractions of the activated dendritic cells and activated mast cells (Fig. 6B). The results from the correlation matrix indicated that the M1 macrophages showed a positive correlation with the CD8^+^T cells and activated memory CD4^+^T cells (Fig. 6C).

### The association between LRP1B mutation and immunoregulator

**Fig. 7:**
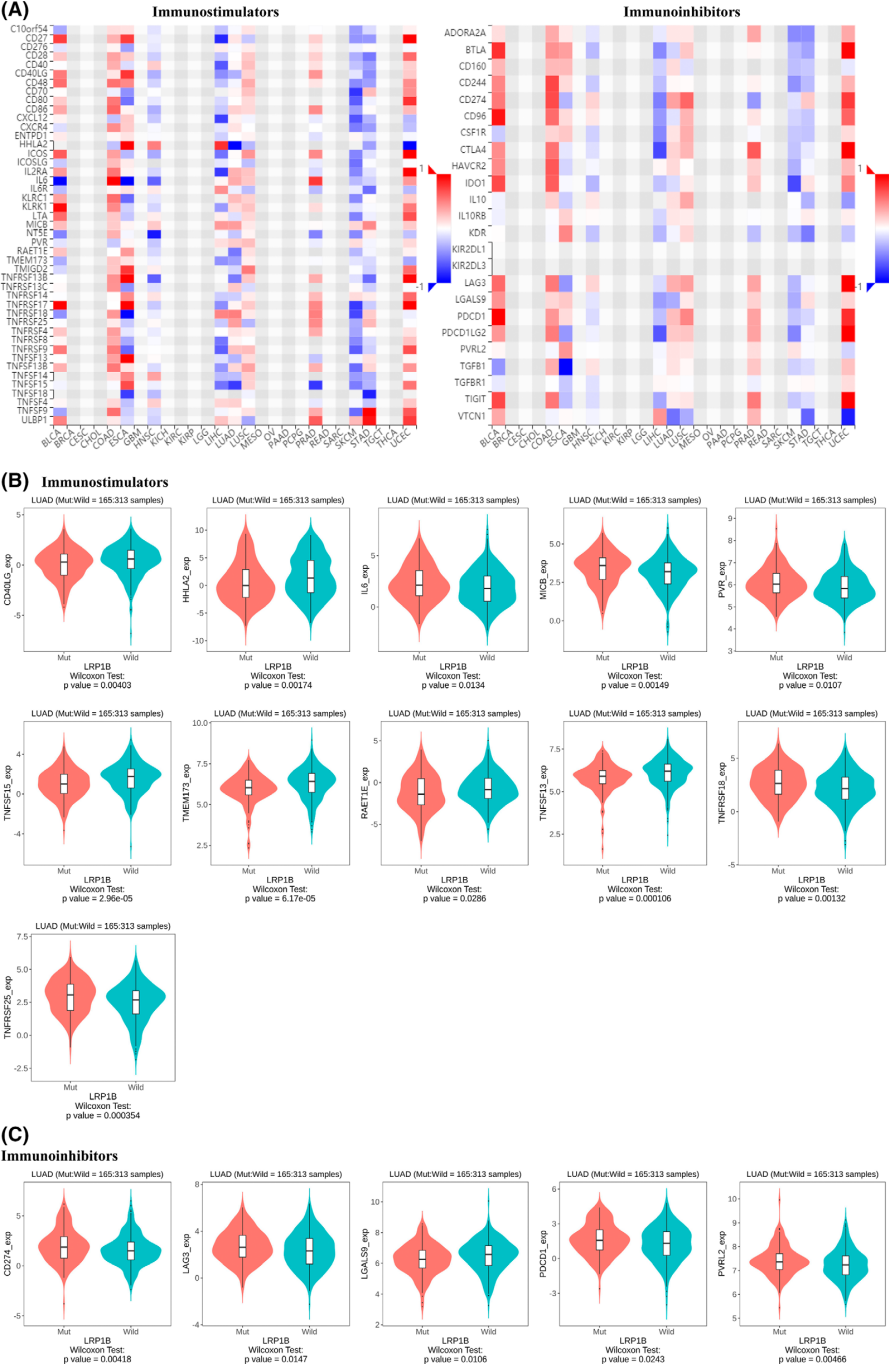
Association between LRP1B mutation and immunoregulators. (A) the heatmap showed the associations between LRP1B mutation and immunostimulators and immunoinhibitors in different cancers, respectively. Integrative analysis between LRP1B mutation with immunostimulators (B) and immunoinhibitors (C).

As shown in Fig. 7, weakened antitumor immunity was observed in the LRP1B‐mut tumors in LUAD. Therefore, the association between the LRP1B mutation and stimulating and inhibitory immune‐regulators was analyzed only in LUAD using the TISIDB database (Fig. 7A).

For the immunostimulators, differences were observed between the LRP1B mutation and LRP1B wild‐type groups in CD40LG, HHLA2, IL‐6, MICB, PVR, TNFSF15, TMEM173, RAET1E, TNFSF13, TNFRSF18, and TNFRSF25 (Fig. 7B). For the immunoinhibitors, compared with the wild group, the levels of CD274 (PD‐L1), LAG3, PDCD1 (PD‐1), and PVRL2 were significantly higher in the LRP1B mutation group; however, LGALS9 was downregulated (Fig. 7C).

### The association between LRP1B mutations and chemokines and receptors

**Fig. 8:**
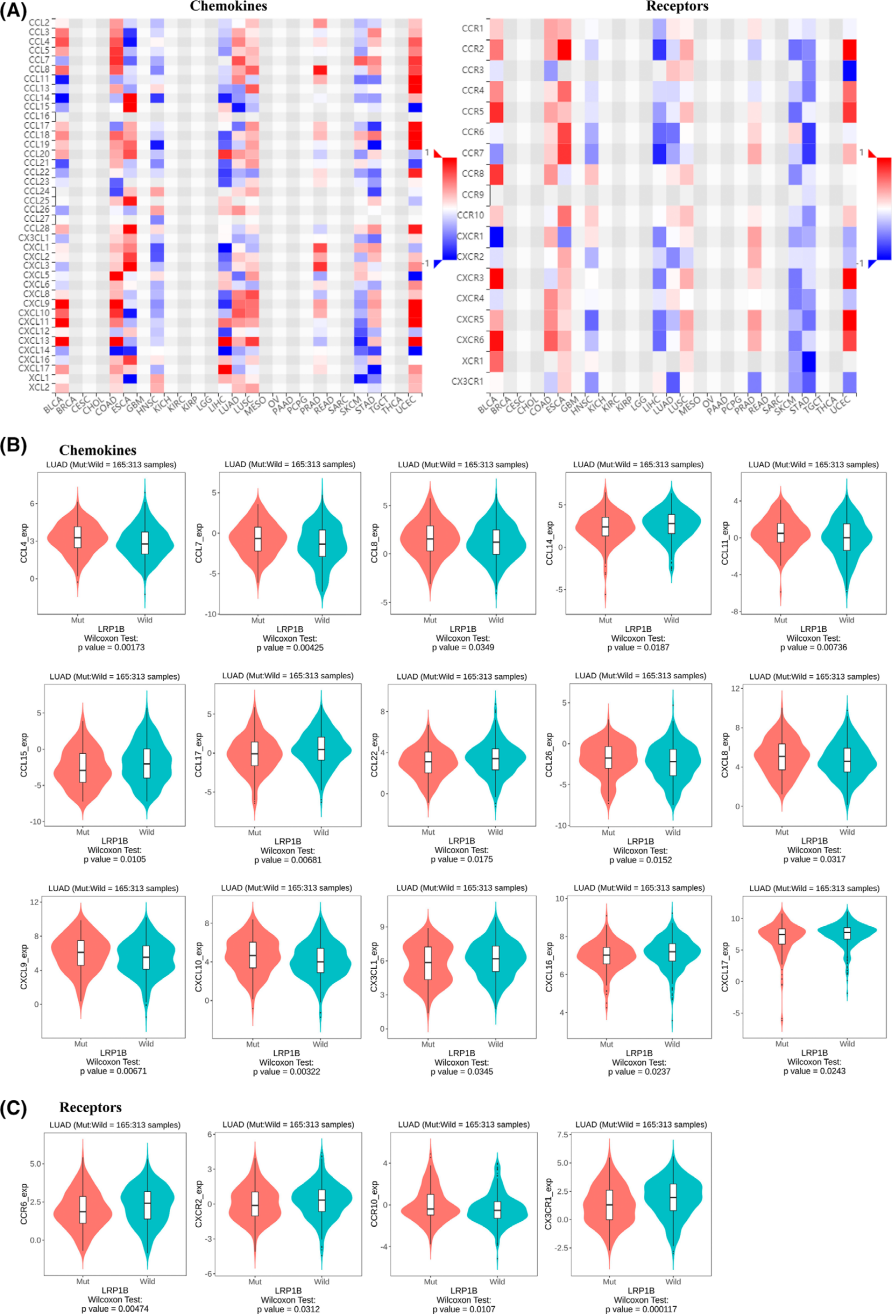
Association between LRP1B mutation and chemokines. (A) the heatmap showed the associations between LRP1B mutation and chemokines and receptors in different cancers, respectively. Integrative analysis between LRP1B mutation with chemokines (B) and receptors (C).

Chemokines and chemokine receptors secreted by or expressed on the surface of both the tumor and immune cells are also involved in tumor immunity. The BioPortal database was also chosen for assessing the correlations between LRP1B mutations and the chemokines and receptors (Fig. 8A). As shown in Fig. 8B, there were obvious differences in the expression levels of some chemokines such as CCL4, CCL7, CCL8, CCL14, CCL11, CCL15, CCL17, CCL22, CCL26, CXCL18, CXCL9, CXCL10, CX3CL1, CXCL16, and CXCL17. For the chemokine receptors, noticeable discrepancies were observed between the two groups for CCR6, CXCR2, CCR10, and CX3CR1 (Fig. 8C).

### Enrichment pathway analysis of the LRP1B mutation

**Fig. 9:**
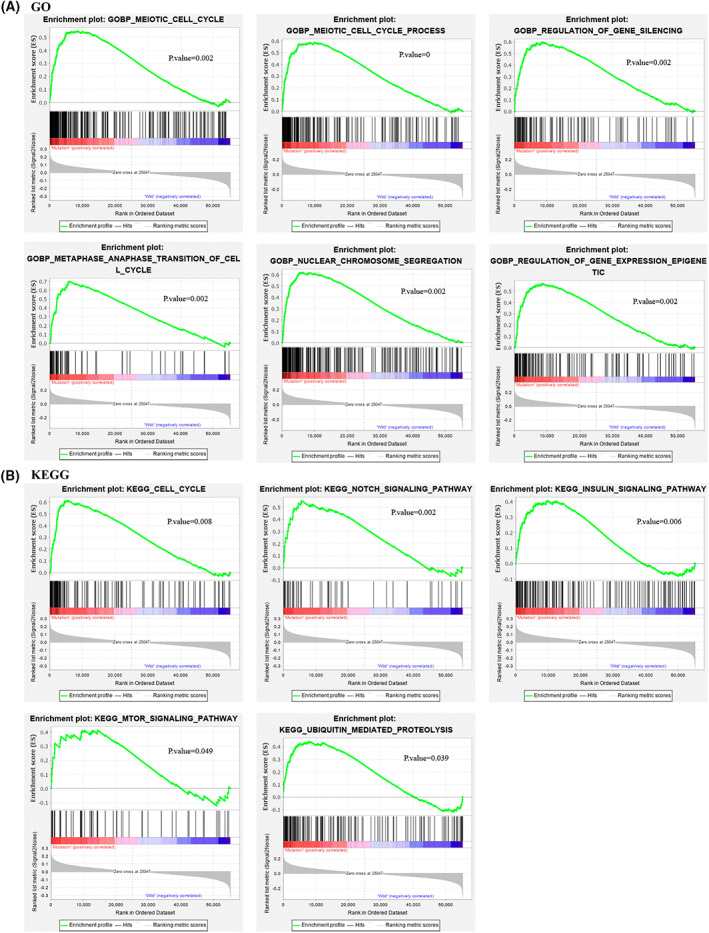
Representative pathways identified by GSEA was performed with the TCGA. Gene enrichment plots performed by functional enrichment of GO biological processes (A) and KEGG pathway enrichment analysis (B) showed that a series of gene sets in LRP1B‐mutant group. The *P*‐value is marked in each plot.

Gene set enrichment analysis (GSEA) revealed that the enrichment of genes related to the regulation of the meiotic cell cycle, meiotic cell cycle process, gene silencing, metaphase anaphase transition of the cell cycle, nuclear chromosome segregation, and regulation of gene expression epigenetics were altered significantly in LRP1B‐mutated samples according to the GO analysis (all *P* < 0.05 and FDR < 0.25, Fig. 9A). Based on the KEGG analysis, pathways that were upregulated significantly in the LRP1B mutant group including the cell cycle, Notch signaling pathway, insulin signaling pathway, mTOR signaling pathway, and Ubiquitin mediated proteolysis (all *P* < 0.05, FDR < 0.25, Fig. 9B). These results demonstrated samples with LRP1B mutation upregulated signaling pathways involved in the growth process of tumors and immune system. The other significant results (*P* < 0.05, FDR < 0.25) of the GSEA analysis are summarized in Table S1.

## Discussion

Next‐generation sequencing techniques offer a powerful approach to discover specific mutations that provide novel‐targeted treatment opportunities for patients with cancer. This has brought about unprecedented advances in personalized treatment decisions, wherein the choice of treatment regimen can be based on individual spliceogenic variants, rather than from results from population‐based studies. NSCLC is characterized by molecular heterogeneity and diversity. In recent years, new molecular‐targeted drugs for genes have produced good results. However, the genomic alterations in NSCLC are complex and NGS can be used to detect numerous novel genes of clinical interest.

In our study, the most frequently mutated genes identified using NGS in the 110 NSCLC patients were EGFR, TP53, LRP1B, FAT1, KMT2D, CREBBP, and RB1. Our results are similar to those reported by a few studies in which NSCLC patients had high rates of EGFR and TP53 mutations [12, 13]. In agreement with a previous study, genetic variations were observed most commonly in TP53 (47.6%), EGFR (41.7%), CREBBP (23.1%), and LRP1B (15.5%) [14]. However, our observed incidences of TP53 and EGFR mutations in NSCLC were slightly higher than those previously reported [14, 15]. In our single‐center study, NSCLC patients also had high frequencies of FAT1, KMT2D, and RB1 mutations, with lower mutation frequencies in other genes. In the Chinese population, these genetic alterations have also been detected in other cancers; however, the mutation frequencies varied greatly [16, 17]. Pathogenic missense mutations were found to be the most frequent mutation subtype, which is consistent with the results of some studies [12, 14]. These differences demonstrate that great molecular heterogeneity was observed in NSCLC.

It is widely accepted that the TMB is a viable biomarker that can predict the survival benefit in NSCLC patients undergoing ICIs treatment [18, 19]. Our results showed that a significantly higher TMB was observed in men and in TP53, LRP1B, KMT2D, CREBBP, and RB1 mutations. Our results are similar to a study conducted by Xiao D, in which men had a noticeably higher TMB than women [20]. While previous studies indicated that there were significant associations between the TMB and EGFR mutations [21], our analysis showed that there were no differences between the EGFR mutant and wild‐type groups. In line with previous observations [22, 23], our analysis also demonstrated that the TMB is associated with TP53 mutations in NSCLC. Moreover, there was evidence that the TMB showed a noticeable increase in cancer samples with LRP1B mutations compared to those with the wild‐type mutations [24, 25], which was consistent with our results. In this study, we observed relationships between the KMT2D, CREBBP, and RB1 mutations and the TMB scores. However, the precise nature of these interactions remains unclear, and further study is recommended.

Several studies have demonstrated that TP53 and EGFR mutations are important factors in the monitoring of the responses to ICI treatment in NSCLC, and the five other genes were chosen for further analysis. The relationship between gene alterations and survival was also explored. This indicated that LRP1B alterations showed a significant correlation with worse OS outcomes, which was in accordance with studies conducted on hepatocellular carcinoma (HCC) [25, 26]. Conversely, a study performed by Chen et al. [24] demonstrated that LRP1B mutations were associated positively with OS in NSCLC and melanoma patients based on data from TCGA database. Notably, in our study, a significant association between LRP1B alterations and DFS was observed. These differences are most likely due to data being obtained from different databases and cohorts, and the gene alterations in the cBioPortal comprising different variables, such as expression levels.

LRP1B, a member of the LDLR protein family, is inactivated mainly in multiple cancers and is considered to be a tumor suppressor gene. In recent years, a few studies have found that LRP1B has a high mutation frequency in malignancies such as lung cancer, HCC, and melanoma [24, 25, 27]. Of note, LRP1B mutation is related to a higher TMB [28, 29]. Consistent with the published studies, in our study, LPR1B is one of most frequently detected mutant genes and significantly higher TMB was also observed in NSCLC patients with LRP1B mutations than those with wild‐type LRP1B. Growing evidence suggests that LRP1B mutations may enhance immune response, and patients with LRP1B mutations exhibit prolonged survival when receiving immunotherapy [24, 30]. Inconsistent results from one study revealed that HCC patients with an LRP1B mutation in peripheral blood had a poor immunotherapy treatment response and inferior prognosis than that those with wild‐type [31]. High TMB usually indicates that there are more neoantigens to activate T‐cell immunity and thus, the immune system will have a higher chance of detecting tumor cells and mounts a stronger anti‐tumor response [32]. Further, TMB is a potential biomarker for ICIs; moreover, patients with a higher TMB benefit frequently from immune treatment [33, 34]. Of course, based on this criterion, not all patients will benefit from immunotherapy.

In subsequent studies, evidence has indicated that the gene status influences the immune microenvironment, which affects the efficacy of ICI therapy. The association between LRP1B mutation and the density of tumor‐infiltrating lymphocytes (TILs) in NSCLC was investigated comprehensively. Previous published data suggested that in HCC, some immune cell infiltration was significantly increased in the LRP1B mutant group, such as naive CD4^+^T cells [26]. In accordance, our data demonstrated that the LRP1B mutation had a positive impact on the levels of immune cell infiltrations, particularly in LUAD. The percentages of B cells, CD4^+^T cells, CD8^+^T cells, Tfh, and M1 macrophages showed significant increases. Tfh cell contributes to the formation, activation, and differentiation of B cells and the M1 macrophages enhance the antitumor function of CD8^+^T cells. Moreover, we found that the LRP1B mutations showed a significant correlation with immunostimulators and immunoinhibitors. Tumor‐associated stromal cells, particularly TILs, which secrete abundant chemokines, also play a critical role in the tumor immune microenvironment. Therefore, we speculated that LRP1B mutation was more likely to improve the tumor immune microenvironment to enhance tumor immunogenicity and antitumor immunity in NSCLC.

Emerging evidence has suggested that LRP1B has several biological functions, including mediating innate adaptive immunity. Our observations also revealed a relationship between LRP1B mutations and the immune response. GSEA revealed that the Notch, insulin, and mTOR signaling pathways were enriched significantly in NSCLC samples with LRP1B mutations. Consistent with a previous study [26], immune response associated signal pathways were enriched in samples with LRP1B mutations. Recent reports have demonstrated that Notch signaling is crucial for T‐cell differentiation and function in cancer [35, 36]. For instance, the activation of Notch signaling promotes IL‐2 secretion and CD25 expression, while the naive CD8^+^T cells differentiate into cytotoxic and memory subtypes, which enhance the development of antitumor immunity [37]. Macrophages are a group of heterogeneous and plastic cells that can be divided into two phenotypes: proinflammatory M1 and anti‐inflammatory M2. The M1 and M2 phenotypes have pro‐ and antitumor functions, respectively. Furthermore, insulin signaling has the potential to mediate macrophage polarization in cancer [38, 39], while mounting evidence supports the role of mTOR signaling in autoimmunity, which is also essential for the activation and proliferation of T‐cells [40, 41]. Together, these reports support the role of the LPR1B mutation in the immune regulation of the TME.

This study had some limitations. First, the patients were recruited from only one clinical center, and the sample size was relatively small. Second, some of the data were collected from several public databases, and no clinical data that assessed LRP1B as a predictive biomarker for NSCLC patients with ICIs treatment, was available. Finally, the mechanisms of LRP1B mutations in tumor immunology remain unclear and require further experimental validation.

## Conclusions

In summary, the genomic mutations underlying NSCLC in a Chinese population were measured using NGS. LRP1B mutations are associated with a higher TMB and with antitumor immune responses. Therefore, it may be a potential biomarker for assessing ICI efficacy.

## Conflict of interest

The authors declare no conflict of interest.

## Author contributions

FX and CL designed the study and collected the data; FX and WQC analyzed the data and made the figures; FBF and JTZ supervised the data collection and directed the r software; RJL reviewed the literatures; FX and WQC wrote the original manuscript; CGS reviewed and revised the manuscript.

## Supporting information


**Table S1.** GSEA analysis of LRP1B mutations.

## Data Availability

The data that support the findings of this study are available on request from the corresponding author.
